# miLBP: a robust and fast modality-independent 3D LBP for multimodal deformable registration

**DOI:** 10.1007/s11548-016-1407-2

**Published:** 2016-06-01

**Authors:** Dongsheng Jiang, Yonghong Shi, Demin Yao, Manning Wang, Zhijian Song

**Affiliations:** Shanghai Key Laboratory of Medical Image Computing and Computer Assisted Intervention, Shanghai, China; Digital Medical Research Center of School of Basic Medical Sciences, Fudan University, Shanghai, China

**Keywords:** Deformable registration, Multimodal image, Similarity measure, Ultrasound

## Abstract

**Purpose:**

Computer-assisted intervention often depends on multimodal deformable registration to provide complementary information. However, multimodal deformable registration remains a challenging task.

**Methods:**

This paper introduces a novel robust and fast modality-independent 3D binary descriptor, called miLBP, which integrates the principle of local self-similarity with a form of local binary pattern and can robustly extract the similar geometry features from 3D volumes across different modalities. miLBP is a bit string that can be computed by simply thresholding the voxel distance. Furthermore, the descriptor similarity can be evaluated efficiently using the Hamming distance.

**Results:**

miLBP was compared to vector-valued self-similarity context (SSC) in artificial image and clinical settings. The results show that miLBP is more robust than SSC in extracting local geometry features across modalities and achieved higher registration accuracy in different registration scenarios. Furthermore, in the most challenging registration between preoperative magnetic resonance imaging and intra-operative ultrasound images, our approach significantly outperforms the state-of-the-art methods in terms of both accuracy ($2.15\pm 1.1 \hbox { mm}$) and speed (29.2 s for one case).

**Conclusions:**

Registration performance and speed indicate that miLBP has the potential of being applied to the time-sensitive intra-operative computer-assisted intervention.

## Introduction

Multimodal deformable registration is widely used in computer-assisted intervention, which aims to identify the correct transformation among different sets of images of the same organ. It is becoming increasingly important to register high-quality preoperative images, such as magnetic resonance imaging (MRI) and computed tomography (CT), with low-quality images captured intra-operatively in real time, such as ultrasound (US), to balance the clinic demand for real-time feedback and accuracy during intervention [1]. Comparing to mono-modal deformable image registration, the definition of the registration objective function, in particular, finding a proper similarity measure, is more challenging in multimodal cases, in which images of different modalities are generated according to different physical principles and are not similar in nature [2]. Approaches using conventional similarity measures, such as mutual information (MI) and normalized mutual information (NMI), tend to fail in the registration between MRI and US [3]. Many methods have been proposed to overcome this difficulty.

The first approach is to calculate a more robust similarity measure directly from the intensity of the two images. Most methods in this category are variants of MI techniques, such as weighting the ${\upalpha }$-MI metric using self-similarity (SeSaMI) [4] and conditioning the MI estimation on similar structures (CoCoMI) [5]. However, current approaches are not practical in terms of implementation effort or computation time.

Another approach is based on simulating US images from MRI. For example, in [6] the authors generate a pseudo-US from an pre-segmented MRI and then use the mono-modal registration method for registration, and in [7, 8] a similarity measure is obtained on the base of the rationale that linear combinations of MRI intensity and gradient magnitude can fit the US intensity by least squares (LC2) [7, 8]. However, these methods require either a preprocessing step to segment the MRI image or the utilization of a large computation for local estimation. The third approach is to map both images to a common space by extracting modality-independent structural descriptor for each voxel from its spatial neighborhood. The rationale is that both modalities image the same anatomical structure, and local geometry information can establish meaningful correspondences. Heinrich et al. have proposed a 6D modality-independent neighborhood descriptor (MIND) [9] based on the local self-similarity principle by measuring the patch distances using the sum of square differences (SSD) between central patch with its six neighboring patches within one image and extended it to a 12D self-similarity context (SSC) [10]. The principle of local self-similarity captures local internal geometric layouts within images. This approach has been successfully used in both CT-MRI registration and MRI-US registration and is proved to outperform recently proposed MI-based approaches [9, 10]. However, the computational effort for calculating and matching a 6D/12D floating-point voxel-wise descriptor for a 3D volume is still very high. To speed up the calculation, the author quantized the 12D SSC into a 60-bit binary string and utilized the Hamming distance between binary strings instead of sum of absolute differences (SAD) between vectors to calculate descriptor’s similarity [10]. But such shortening technique still remains inefficient in the sense that first computing a high-dimensional floating-point descriptor and then shortening it still involves a substantial amount of computation. Furthermore, due to the scale difference of the patch distances for different modalities, a normalization step is needed to calculate the original 6D/12D floating-point descriptor. In addition, the absolute patch distance is easily affected by the noise in the patches, and an extra step is required to compensate the noise effect by dividing the local variance [9].

**Fig. 1:**
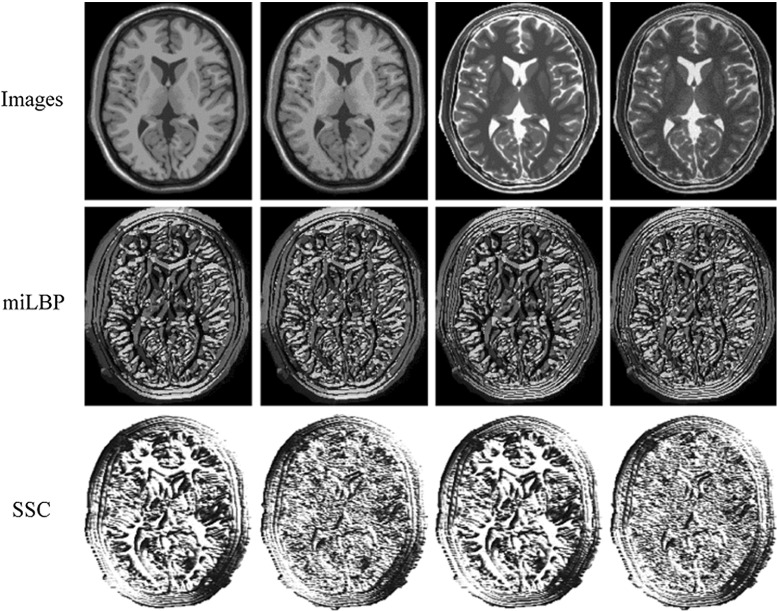
The miLBP and SSC descriptor images for clean and degraded T1 and T2 images. The *first row* from *left to right*: the original T1 image, T1 image with 3 % noise and 40 %, the original T2 image, T2 image with 3 % noise and 40 % intensity nonuniformity. The *second and third rows* are the corresponding descriptor images for 2D miLBP and 2D SSC. For 2D miLBP, we used 8 neighbors for miLBP clacluation and map the 8-bit binary strings to integer values in the range of [0, 255]. For the 4D vector-valued descriptor of 2D SSC, we only show the first-dimensional for displaying

Recently, descriptors consisting of binary strings, such as local binary pattern (LBP) [11] and binary robust independent elementary features (BRIEF) [12], have drawn significant attention and have achieved excellent performance in various applications, such as recognition and classification [13]. Both descriptors are computed by pairwise comparing the intensity the encoded central pixel with its neighbors. Inspired by the promising results of local self-similarity-based descriptors in medical image registration and the fast and robust property of binary descriptors, in this paper, we present a novel robust and fast modality-independent binary descriptor by directly computing a binary string for each voxel based on local self-similarity principle.

Compared with previously proposed local self-similarity-based descriptors MIND/SSC, the proposed descriptor can be computed more efficiently by simply thresholding the voxel distances between the central voxel and its neighbors in a small smoothed image patch. The most straightforward voxel distance measure between two voxels, SAD, is used. This approach naturally forms a 3D LBP, and thus, we refer to the resulting descriptor as modality-independent LBP (miLBP). Furthermore, the binarized voxel distance is robust to image degradations such as noise and intensity nonuniformity that widely present in medical images when using the standard deviation as the adaptive threshold. Figure 1 shows the 2D miLBP and the 2D SSC descriptor images of a pair of clean T1 and T2 images and the degraded ones with 3 % noise and 40 % intensity nonuniformity. As shown in Fig. 1, for the original images, both descriptors can extract discriminative features, such as corner, edges and textures across modalities, but when the images are degraded by noise and intensity nonuniformity, the features of SSC are blurred severely, while the miLBP features are only slightly influenced. miLBP can also be compared very efficiently using the Hamming distance, which can be calculated extremely fast on modern CPUs that support the SSE4.2 instruction set containing dedicated functions for performing an XOR or bit count operation [14].

We tested the general applicability of our method with several experiments. We first applied miLBP to artificial images representing CT and MRI images with different noise level. Next, we performed deformable registration between different MRI modalities and between CT and MRI from BrainWeb [15] and RIRE [16]. Finally, we performed preoperative MRI and intra-operative US images registration for image-guided neurosurgery (IGNS) from BITE [17].

In the next section, we give the detailed explanation of miLBP. In “Experiments” section, the validation results are presented. Finally, the results are discussed in “Discussion” section and conclusions are given in “Conclusion” section.

## Methods

This section provides a basic description of LBP and then explains the detail of our proposed descriptor.

### LBP

**Fig. 2:**
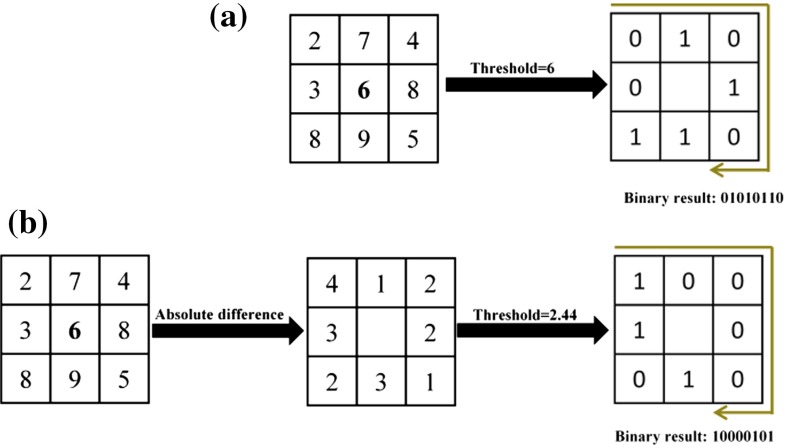
Illustration of obtaining the LBP (**a**) and the miLBP (**b**) in 2D

The LBP operator, proposed by Ojala et al. [11], is a very simple and efficient local descriptor for describing textures. For each pixel in a 2D image, the operator labels the pixel’s certain neighborhood with 0 or 1 by thresholding with the value of the center pixel and then calculates a new value for the pixel by multiplied by powers of two. The output value of the LBP operator for a block of 3 $\times$ 3 pixels can be defined as follows:

1 $$\begin{aligned} \hbox {LBP}_{8} =\sum _{i=1}^8 s\left( {g_i -g_0 } \right) 2^{i-1},\quad s\left( x \right) = \left\{ \begin{array}{ll} 1, &{} \quad x\ge 0 \\ 0, &{} \quad x<0 \\ \end{array} \right. \end{aligned}$$

where $$g_0 $$ corresponds to the intensity of the central pixel and $$g_i $$ is the intensity of the ith neighboring pixel. The signed intensity difference $$\left( {g_i -g_0 } \right) $$ is unaffected by the change in mean luminance, which provides the grayscale invariance to the operator. An example of obtaining LBP is shown in Fig. 2a.

### miLBP

To get a local self-similarity-based 3D binary descriptor, we consider a $3\times 3\times 3$ patch in a 3D volume and binarize local self-similarity into 0 or 1 by thresholding the voxel distances between the central voxel and its 26 neighbors. In other words, we get 1 for a high response that those neighboring voxels are significantly different from the central voxel and get 0 for a low response for everything that is similar. The signed intensity difference used by BRIEF and LBP is not suitable for multimodal images, because corresponding location may have inverted intensity transitions in two different modalities. For example, the intensity of white matter is higher than that of gray matter in T1 image, but lower in T2 image. The inverted intensity transitions will lead to inverted 0/1 coding, which make it impossible to extract a similar descriptor value.

To properly binarize the distance across different modalities and positions, the most important issue is to find a proper and unbiased threshold to divide high and low responses. Obviously, a user-defined or a universal threshold could not work, and the threshold must be adapted to image modalities and local intensity distribution.

In this study, we exploit the local intensity statistics (local standard deviation) of patches for extracting stable miLBP features. An example of obtaining 2D miLBP is shown in Fig. 2b. The standard deviation can work well as an adaptive threshold for different modalities because if two modalities have the same local internal geometric layouts, they have the same local distance pattern consisting of 26 voxel distances, but the scale of the distances might be different for different modalities; in MIND/SSC, they normalized each vector-valued descriptor into [0, 1] to eliminate the scale difference. The local standard deviations are linearly proportional to the scale of the distance, so we naturally obtain a scale invariant descriptor using this adaptive threshold. Furthermore, the standard deviations can statistically reflect the significances of the voxel distance and the high response is rarely broken up by the image degradations such as noise and intensity nonuniformity, which enhances the robustness of miLBP. Local standard deviation was also used in previous studies to enhance the robustness of LBP methods. In [18], the authors used local standard deviation as an adaptive threshold to compute a local adaptive ternary pattern (LATP). A similar strategy was also used to extract robust acoustic features from spectrograms of environmental sounds to cope with fluctuations in pixel values [19]. To further avoid the noise sensitivity of single voxels, we adapted the strategy used in BRIEF that applies a pre-smoothing step on the patch using Gaussian kernel [11]. The final miLBP is described by the following equation:

2 $$\begin{aligned} \hbox {miLBP}=\sum _{i=1}^{26} s(|g_i -g_0 |)2^{i-1},\quad s\left( x \right) =\left\{ \begin{array}{ll} 1,&{} \quad x>\delta \\ 0,&{} \quad x\le \delta \\ \end{array} \right. \end{aligned}$$

where $g_0$ corresponds to the intensity of the central voxel and $\hbox {g}_{\mathrm{i}}$ is the intensity of the 26 surrounding voxels. The absolute intensity difference $(|g_i -g_0 |)$ combined with the standard deviation $\delta$ as the threshold is used to compute the labels. Thus, $\delta$ must be calculated for every central voxel using all 27 voxels within the patch. Fortunately, the calculation can be simplified without loss of accuracy using box filters, following the ideas of guided image filtering [20]. By incorporating this simplification, the complexity of calculating $\delta$ is greatly reduced and becomes independent of the patch size such that miLBP can still be computed and stored very efficiently with only a few operations and a few bits of memory.

**Fig. 3:**
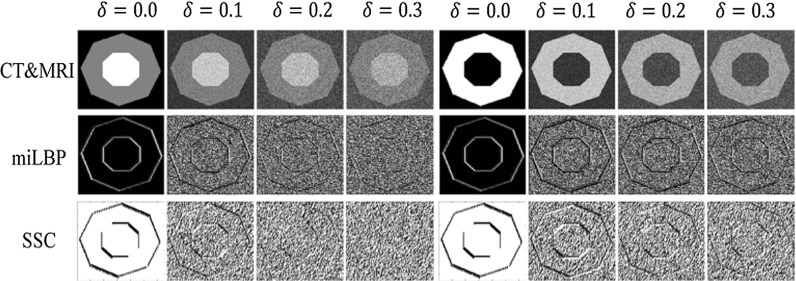
Artificial images representing CT and MRI with different levels of noise and their corresponding descriptor images of miLBP and SSC. The *left four* images are the CT images. For 2D SSC, we only show the first-dimensional values of the 4D vector-valued descriptor

**Fig. 4:**
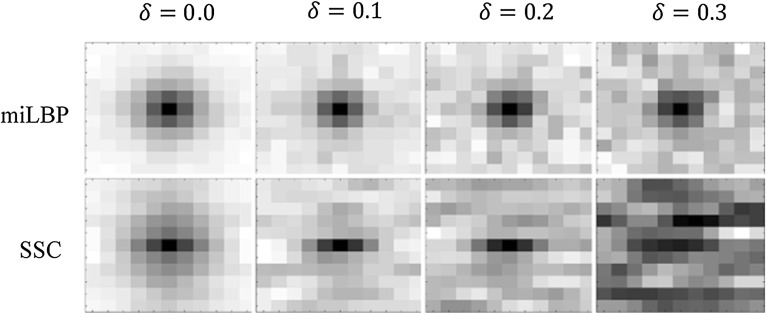
Illustration of the influence of the noise on the two similarity measures. Dissimilarity values at each noise level are plotted with respect to the translation of the CT image in the *x*, *y* direction in a range of ±5 pixels. The images were aligned at 0 displacements, where the dissimilarity value between the fixed MRI image and the moving CT image should be smallest

## Experiments

We conducted several experiments to test the general performance of miLBP for multimodal image registration, ranging from artificial image to clinic data. We first compared the robustness between SSC and miLBP using artificial images representing CT and MRI with different levels of noise. Then, we performed 3D multimodal deformable registration between different MRI modalities and between CT and MRI from BrainWeb and RIRE. Finally, we registered the preoperative MRI and the intra-operative US images from BITE.

In the following experiments, we employed the same registration framework used by SSC, which is a recently proposed Markov random field (MRF)-based discrete optimization strategy called Deeds [21]. We implemented miLBP and SSC in the same framework using the C++ language to enable directly comparison, and we only compared SSC with miLBP in this study because SSC has been proven superior to recently proposed MI-based approaches [10], such as conditional MI (cMI) [22] and entropy image [23]. Compared with continuous optimization, which is prone to local minima and requires an analytical or a numerical derivative of the cost function, Deeds uses a block-wise parametric transformation model with belief propagation on a tree so that it can obtain the global minima without derivative calculation. More details on Deeds can be found in the original paper [21]. We used the original parameter setting for SSC: a patch size of $3\times 3\times 3$ voxels and a distance between neighboring patches of 2 voxels and quantizing the 12D descriptor to a single integer value with 64 bits for the similarity calculation. The similarity term for miLBP and SSC is the Hamming distance between the corresponding descriptors, and 50 samples were randomly selected to calculate the similarity at each control point. An optimal regularization parameter, ${\upalpha }$, for SSC is 0.5. The best ${\upalpha }$ for miLBP we found is 0.7. Other parameters of Deeds are listed in the corresponding subsections, which were selected according to the displacement ranges of different tasks.

### Artificial images

Before registration experiments, we first calculated the descriptor image and the similarity values of miLBP and SSC on artificial images representing CT and MRI with different noise levels. We created one artificial 2-D multimodality image pair by using the method in [22], as shown in Fig. 3, which are inspired by a slice through a lower limb in CT and MRI, picturing background, muscle and bone, consisting of a dark background (*I* = 0) and two concentric octagons: the soft tissue, represented by a larger octagon with a medium intensity (*I* = 200) for CT and MRI, and the bone, represented by a smaller octagon with high intensity in the CT image (*I* = 400) and dark (*I* = 0) in the MR image. At first, we normalized the intensity of the two images into the range of [0, 1]. We then added Gaussian white noise of mean 0 and standard deviation $\delta \,=\,0.0,\,0.1,\,0.2,\,0.3$ to the two images. The corresponding degraded images and their descriptor images of 2D miLBP and 2D SSC are also shown in Fig. 3. We can see the descriptor images are also degraded along with the increase in the noise level. When $\delta$ increases to 0.2 or larger, the octagon structures become invisible for SSC of the CT image. miLBP shows a strong robustness property with octagon structures visible for all the degraded images.

The influence of the noise on the similarity measures is shown in Fig. 4. At each noise level, we moved the CT image in the *x*, *y* direction in a range of $\pm 5$ pixels, and at each displacement, the dissimilarity value between the moved CT image and the MRI was calculated. We can see miLBP can still work as a proper similarity measure for all the noise levels, but SSC fails when the noise level increases to 0.3.

**Fig. 5:**
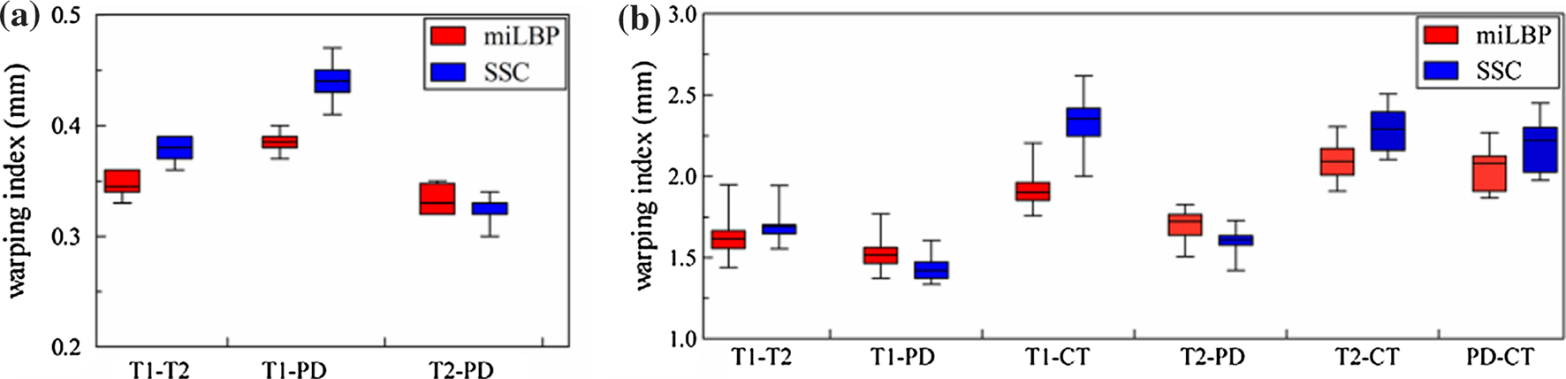
The warping index for different pairwise registrations using miLBP and SSC. For all the registration tests, the *p* value are lower than 0.05, which indicate the statistical significance. **a** BrainWeb. **b** RIRE

**Fig. 6:**
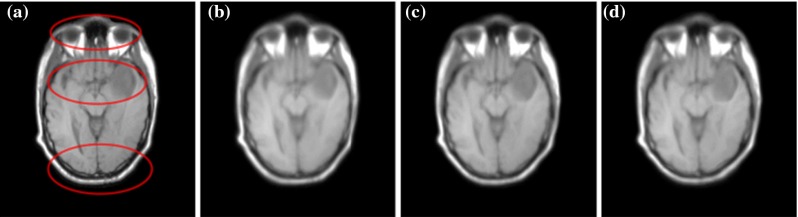
Average of the results obtained over all 20 registrations after displacement field distortion. **a** Original. **b** Average warped. **c** Average miLBP. **d** Average SSC

### BrainWeb and RIRE

The BrainWeb database consists of simulated T1, T2 and PD brain MR images of $181\times 217\times 181$ voxels with a 1 mm voxel size in each dimension, calculated from a single phantom, and provides a gold standard for different similarity measure tests. The training set of RIRE database includes T1, T2, PD and CT images with ground truth transforms and has also been used in many other registration evaluations [23]. In this study, we normalized all four modalities in RIRE into the coordinates of the PD image (with rectification) with a voxel spacing of $1.26\times 1.26\times 4.1 \hbox { mm}$ according to the given transforms. We performed pairwise multimodal deformable registration on these two databases. We first generated 20 smooth deformation fields by moving every node in three dimensions by uniformly distributed random numbers. Next, we obtained the fixed image by deforming one of the volumes using these 20 displacement fields and registered the moving image to it. For validation, we calculated the warping index to estimate the registration error. The warping index is the root-mean-square of the displacement error of all image voxels. The initial warping index before registration for Brianweb and RIRE was $2.71\pm 0.51$ and $3.25\pm 0.25 \hbox { mm}$, respectively. For Brianweb, we used one modality with 0 % noise and 0 % intensity nonuniformity as the fixed image and the other modality with 3 % noise and 40 % intensity nonuniformity as the moving image for registration. The following parameters were set for the Deeds in this registration experiments: three scales of control point spacing of $\{10,\, 8,\, 4\}$ voxels and a dense displacement search range of $\{4,\, 4,\, 3\}$ voxels (with a spacing of $\{2,\, 1,\, 1\}$ voxels). The results are shown in Fig. 5. In the registration of T2-PD in both databases and T1-PD in RIRE, the accuracy of miLBP is lower than SSC, but for the other registration tests, the accuracy of miLBP is higher than SSC.

As Deeds uses inverse consistent transformations to obtain inverse consistent mappings, we can get the backward displacement fields to correct the deformed fixed images. In the registration between the deformed T1 image and the CT image from RIRE database, we average the corrected T1 images and the results are shown in Fig. 6. The average image obtained from miLBP is clearly sharper than the one obtained from SSC, indicating higher registration accuracy of miLBP. The detailed improvements can be seen in the parts marked with red circles. In the upper red circle, the eyes from SSC are shrunken and blurred severely, but it is clear in miLBP as the original image. In the middle red circle, the tumor from SSC is blurred. In the lower red circle, the boundary from SSC is inflated compared with the original image.

### Clinical MRI and US image

We further evaluated our method with clinical MRI and US images in the context of IGNS. The registration task consisted of matching preoperative MRI patient volumes to intra-operative US volumes. A set of 13 pairs of preoperative MRI and pre-resection 3D US images of BITE database was used. In the database, on average, 27 corresponding anatomical landmarks were selected for each pair of images, which were used for the evaluation of the deformable image registration by computing the mean target registration error (mTRE). All images were resampled to the isotropic 0.5 mm voxel size. The tracking information was used to perform the initialization, and then the MRI volumes were cropped to the same size as the corresponding US volumes. This initialization was also used in [4, 5, 8], enabling a direct comparison of different methods. Although SSC reported an accuracy of $2.12\pm 1.29 \hbox { mm}$ in [10], its initialization was different. Thus, we rerun the method for a direct comparison.

The following parameters were used for the Deeds in this registration experiments: three scales of control point spacing of $\{12,\, 10,\, 8\}$ voxels and a dense displacement search range of $\{8,\, 5,\, 4\}$ voxels (with a spacing of $\{2,\, 2,\, 1\}$ voxels). The results of miLBP and SSC and results reported in recent published studies are listed in Table 1. The minimum error in each row is highlighted with bold letters.

**Table 1:** A list of currently published results in [4, 5, 8], and results using miLBP and SSC

Case#	Initial	miLBP	SSC	SeSaMI	CoCoMI	LC2
1	6.30	1.79	$\mathbf{1.69}$	1.82	3.22	1.64
2	9.38	2.42	$\mathbf{2.37}$	2.54	3.03	2.43
3	3.93	$\mathbf{1.61}$	1.65	1.96	2.17	1.91
4	2.62	$\mathbf{1.94}$	1.98	2.59	2.18	2.26
5	2.30	1.85	1.88	$\mathbf{1.73}$	2.20	2.20
6	3.04	2.40	2.59	$\mathbf{1.94}$	2.13	2.52
7	3.75	$\mathbf{1.98}$	2.10	2.91	2.00	3.64
8	5.09	2.40	2.42	2.52	$\mathbf{2.18}$	2.65
9	2.99	$\mathbf{1.58}$	1.83	2.74	2.04	2.09
10	1.52	1.88	2.05	$\mathbf{1.35}$	2.48	1.76
11	3.70	2.42	2.80	2.78	$\mathbf{2.16}$	2.45
12	5.15	3.20	3.55	2.91	$\mathbf{2.64}$	3.71
13	3.78	2.52	2.49	2.16	$\mathbf{2.07}$	2.76
Avg.	$4.12\pm 1.5$	$\varvec{2.15\pm 1.1}$	$2.26\pm 1.2$	$2.30\pm 1.3$	$2.35\pm 1.2$	$2.46\pm 0.6$
Avg. time	$\textendash$	$\mathbf{25.4\,s}$	35.2 s	$\approx$120 min	$\approx$10 min	270 s

The last two rows show the average mTRE and time of all cases

The minimum error in each row is highlighted with bold letters

**Fig. 7:**
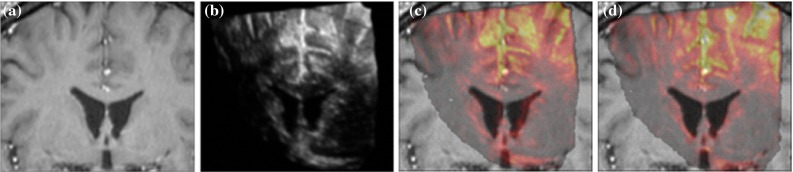
Deformable MRI-US registration results of case 1 using miLBP. **a** MRI slice. **b** The corresponding US slice before registration. The corresponding US slices before and after deformable registration shown in pseudo-color are overlaid on the MRI image in **c** and **d**, respectively

We also conducted the same experiments using SAD that was also implemented in the same registration framework for comparison. SAD did not reduce the initial mTRE, so the results are not listed here. Comparing the average mTRE of the 13 cases shown in Table 1, it can be observed that miLBP achieves the best accuracy of 2.15 mm. The *p* value of statistical significance of the improvement of miLBP over SSC obtained using paired Student’s *t* test is 0.02, which shows that miLBP significantly outperforms SSC and the other methods. SSC and miLBP yield the two highest accuracies, which indicates that a dense local self-similarity-based similarity measure with Deeds is more suitable for deformable registration of MRI and US than other frameworks. The biggest mTRE after initialization is 9.38 mm, and it is reduced to 2.42 mm by deformable registration with miLBP.

In terms of registration time, as different authors used different platforms, the time comparisons with recent published studies are coarse, but the magnitude difference is still convincing. Recently published studies SeSaMI and CoCoMI, which were adapted from MI, require several minutes to several hours to complete per case. LC2 also requires several minutes to complete even with GPU implementation. In our implementation, the average time for SSC is 35.2 s, and miLBP only need 25.4 s, which is the fastest. Considering the time for descriptor computation, for a volume with size of $197\times 212\times 175$, miLBP descriptor can be calculated in only 1.5 s, whereas the time required by SSC is 3.0 s.

Figure 7 shows an example of the registration problem and the resulting alignment using miLBP. An improvement can be observed when comparing images before and after deformable registration. A clearly improved alignment of the ventricles and the gyri is visible after deformation registration.

## Discussion

We have proposed a novel modality-independent neighborhood binary descriptor, miLBP, based on the principle of local self-similarity. The descriptor can be computed vary fast and can robustly map different modalities to a common space. miLBP can be treated as a new variant of LBP. To the best of our knowledge, miLBP is the first 3D LBP intended for multimodal 3D volumes registration. Different from previous local self-similarity-based descriptors with floating-point vector values, we first proposed a binary local self-similarity-based descriptor. After the local threshold is obtained, the 26-bit descriptor can be computed with a few atomic operations without extra steps of normalization and noise estimation. Besides, this bit string descriptor makes it possible to directly compute similarity using the Hamming distance. In all, our proposed descriptor can be computed and matched vary efficiently compared with MIND/SSC.

In the experiment, we have shown that miLBP was more robust than SSC in extracting local geometry features across modalities and achieved higher registration accuracy in different registration scenarios. In experiment 3, we have conducted the most challenging registration between preoperative MRI and intra-operative US. Compared to the recently proposed specialized methods, our approach achieves the best results in terms of both accuracy and speed. We also compare the computation time between miLBP and SAD, in the same registration framework for the BITE database. The average time was 20.1 and 25.1 s for SAD and miLBP, respectively. miLBP only needs approximately five more seconds than SAD, which is usually considered as the most simple similarity measure, and this is due to the fast calculation of miLBP and the Hamming distance. This finding further indicates that miLBP has the potential of being applied to the time-sensitive intra-operative US-guided intervention with a more efficient GPU implementation.

Based on the assumption that the same anatomical structure presents in both modalities, a common limitation of all local self-similarity- based descriptors is that they might not be applicable when the structures shown in the two images are different. Future improvements of this descriptor might be possible by considering circle neighborhood or other thresholding strategy.

## Conclusion

We have presented a new binary modality-independent descriptor, miLBP, based on the principle of local self-similarity, for deformable multimodal image registration. We have shown that miLBP outperformed the state-of-the-art similarity measures in terms of both speed and accuracy in various application contexts.
